# Microbiological Profile and Antimicrobial Susceptibility Pattern of Uropathogens Isolated From Pregnant Women Attending a Tertiary Care Hospital in Central India

**DOI:** 10.7759/cureus.70798

**Published:** 2024-10-03

**Authors:** Girish Patil, Divya Patil, Ashwini Patil, Sunanda Shrikhande

**Affiliations:** 1 Microbiology, All India Institute of Medical Sciences, Raipur, IND; 2 Microbiology, Government Medical College and Hospital, Nagpur, IND

**Keywords:** beta lactamases, escherichia coli, fluconazole resistance, non-albicans candida, pregnancy, uti

## Abstract

Introduction: Urinary tract infections (UTIs) are more common in females than males, and during pregnancy, frequency is increased due to hormonal changes affecting the anatomy and physiology. If not treated on time, complications may develop in both the mother and fetus. This study aimed to analyze the microbiological (bacterial and yeast) profile of the isolated uropathogens and their antimicrobial susceptibility.

Methods: Aseptically collected urine specimens were processed by the standard loop method. Identification of uropathogens was done by standard microbiological tests, and antimicrobial susceptibility was performed as per the Clinical and Laboratory Standards Institute (CLSI) guidelines.

Results: Out of 400 study participants, UTI was detected in 26.75% (107/400) of cases. Overall, *Escherichia coli* (42.99%, 46/107) was the most frequently isolated uropathogen followed by *Candida* species (20.56%, 22/107), whereas, among gram-positive bacteria, *Enterococcus faecalis* (7.47%, 8/107) was the commonest. Among *Enterobacterales*, extended-spectrum beta-lactamase (ESBL) and AmpC beta-lactamase (AmpC) production was observed in 19.35% (12/62, eight *E. coli* and four *Klebsiella*) and 6.45% (4/62, all *E. coli*) isolates, respectively. However, all the gram-negative organisms showed 100% (n=70) sensitivity to carbapenems. Among staphylococci, 50% (2/4) of isolates were methicillin-resistant, and high-level aminoglycoside resistance (HLAR) was observed in 75% (6/8) *of Enterococcus faecalis *isolates, but all were sensitive to vancomycin, linezolid, and nitrofurantoin. Among *Candida* species, non-albicans *Candida* (NAC) species (59%, 13/22) outweigh the number of *Candida albicans* (41%, 9/22), with *Candida tropicalis* (22.72%, 5/22) being the commonest NAC. All the *Candida* species were susceptible to voriconazole, whereas fluconazole resistance was observed in 31.81% (7/22) of *Candida* isolates, with a higher percentage in NAC species (22.72%, 5/22) than in *Candida albicans* (9.09%, 2/22).

Conclusions: UTIs in pregnancy are caused by a spectrum of both gram-negative bacilli and gram-positive cocci. Moreover, there is a rise of both *Candida albicans* and NAC species as uropathogens, with the emergence of fluconazole resistance. There is an increased prevalence of asymptomatic bacteriuria in pregnancy, which necessitates the screening of pregnant females for UTI using culture and antimicrobial susceptibility testing.

## Introduction

Urinary tract infections (UTIs) are one of the common diseases affecting pregnant females. About 50% of all women experience UTIs once in a lifetime [1]. In pregnancy, various anatomical and physiological changes increase the susceptibility to infections [2]. UTI during pregnancy can present as either asymptomatic bacteriuria (ASB) or symptomatic UTI. ASB is defined as the growth of significant bacteria (>10^5^ colony-forming unit (CFU)/ml) in the urine culture of patients without any symptoms suggestive of UTI [3]. 

Pregnancy can progress from ASB to symptomatic UTI including cystitis (40%) and pyelonephritis (20-30%). Pyelonephritis during pregnancy is known to be associated with adverse outcomes like prematurity, intrauterine growth retardation, decreased birth weight, and increased mortality rates of a fetus. In addition, complications can occur in mothers like anemia, pre-eclampsia, eclampsia, chronic pyelonephritis, and, sometimes, renal failure. Hence, diagnosis in the early stages and its prompt treatment are essential [4]. The Infectious Diseases Society of America (IDSA) recommends screening of bacteriuria in asymptomatic patients as well as its treatment [5]. The randomized controlled trials have demonstrated that treatment of ASB during pregnancy will decrease the risk of developing pyelonephritis from 20-35% to 1-4% and the risk of decreased birth weight in newborns from 15% to 5% [6].

The commonest pathogen isolated from UTI cases is *Escherichia coli* among bacteria and *Candida* species among yeast. Candiduria in females is a frequent finding due to its colonization in genital regions, and pregnancy increases the risk for its development. Previous studies reported a predominance of *Candida albicans*, but in recent years, there has been the emergence of non-albicans *Candida* (NAC) species. These species reporting increased resistance to antifungals are difficult to eradicate from the urinary tract [7]. In pregnancy, there is a problem of safety and selection of antimicrobials during pregnancy. Antimicrobial agents like nitrofurantoin, fosfomycin, and β-lactam antibiotics are considered safe in pregnancy [8]. However, there are limitations in the choice of antimicrobials due to the emergence of antimicrobial resistance like the production of the extended-spectrum beta-lactamases (ESBL) by *Enterobacterales* and methicillin resistance in *Staphylococcus aureus* [9].

This research was conducted to estimate the prevalence of symptomatic and asymptomatic UTIs during pregnancy and to analyze the microbiological (bacteria and yeast) profile and pattern of antimicrobial sensitivity of the isolated uropathogens.

## Materials and methods

Study design and duration

This descriptive cross-sectional study was conducted for two years from December 2017 to November 2019 at the Government Medical College and Hospital, Nagpur, India.

Patient consent and ethical approval

This study was approved by the Institutional Ethics Committee of Government Medical College and Hospital, Nagpur (approval number: 1026). After obtaining written consent, participants were explained about the study, and the required clinical history of each patient was noted and specimens were collected.

Inclusion and exclusion criteria

Inclusion Criteria

Pregnant women in all the trimesters, who visited the outpatient department and were willing to participate, were included in the study irrespective of urinary symptoms (fever, loin pain, increased frequency of micturition, urgency, burning micturition, dysuria, etc.).

Exclusion Criteria

Pregnant women, who were not willing to participate, who have active bleeding or discharge per vagina, and who had taken antibiotic treatment in the previous two weeks, and inpatient pregnant females were excluded from the study.

Specimen collection and transport

All the participants were instructed for aseptic precautions, and urine specimens (mid-stream) were collected in sterile uricol containers. Specimens were immediately transferred to the microbiology laboratory and processed within an hour. In case of delay, they were refrigerated at 4°C and processed within 24 hours [10].

Processing of Specimens

Urine specimens were cultured on cysteine-lactose-electrolyte-deficient (CLED) agar and Sabouraud dextrose agar (SDA) with the help of a sterile disposable inoculating loop which holds 0.001 ml of urine (semi-quantitative method). The culture plates were kept in an incubator for 24 hours, aerobically, at 35-37°C. The number of CFU was multiplied by 100 to estimate the number of microorganisms per milliliter of urine specimen [10]. ASB is defined as the growth of >10^5^ CFU/ml of the same species on two consecutive cultures. For symptomatic patients, pure growth of >10^3^ CFU/ml of mid-stream urine in a single culture was considered a significant bacteriuria. The growth of two or more organisms was considered as contamination [11].

Identification of Uropathogens

The bacterial identification was based on colony morphology, gram stain, and biochemical tests [10]. The identification of *Candida* isolates was done by colony characteristics on SDA and HiChrom *Candida* agar, gram stain, germ tube test, morphological appearance by Dalmau technique using cornmeal agar, sugar fermentation, and assimilation tests [12]. 

Antimicrobial susceptibility testing

Antimicrobial susceptibility testing was done using the Kirby-Bauer disk diffusion method according to the Clinical and Laboratory Standards Institute (CLSI) 2017, with appropriate quality control [13]. For ESBL detection, double-disk synergy testing was performed [13], and AmpC beta-lactamase (AmpC) production was detected by using a disk approximation test [14].

Antifungal susceptibility testing was done by disk diffusion method (CLSI M44-A2) for fluconazole (25 mcg) and voriconazole (1 mcg), using Mueller-Hinton agar with glucose (2%) and methylene blue (5 μg/ml), with appropriate quality control [12,15,16]. The minimum inhibitory concentration (MIC) of fluconazole was calculated by epsilometer test (E-test) for *Candida* isolates resistant to fluconazole by disk diffusion [12,16].

Statistical analysis

Collected data were entered and analyzed in Microsoft Excel. The p-value was calculated using the chi-squared test (<0.05 was taken as statistically significance). The results of antimicrobial susceptibility tests were expressed as percentages.

## Results

In this study, a total of 400 pregnant females were included. Significant bacteriuria was present in 26.75% (107/400) of cases. Out of these, 41.12% (44/107) were symptomatic and 58.87% (63/107) were asymptomatic.

**Figure 1:**
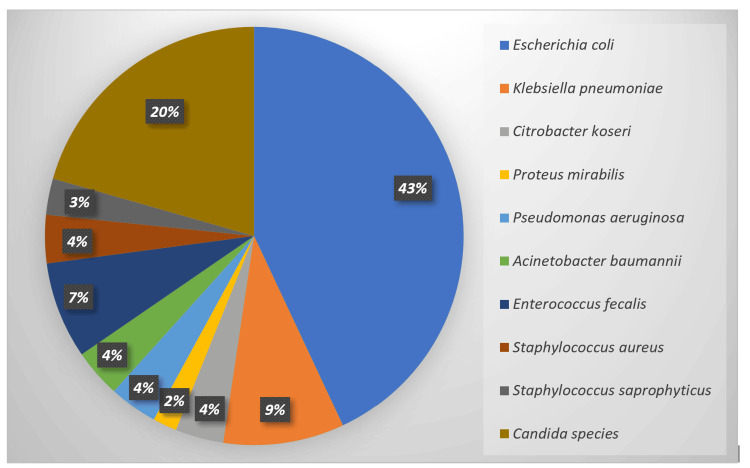
Microbiological profile of uropathogens isolated from pregnant females with significant bacteriuria

UTI was more frequently observed in the third trimester (52.33%, 56/107) (p=0.47) and in primigravida (54.20%, 58/107) (p=0.71). Similarly, we observed increased prevalence in pregnant women belonging to the age group of 26-30 years (65.42%, 70/107) (p=0.05), followed by 31-35 years (23.36%, 25/107). Overall, *Escherichia coli* (42.99%, 46/107) was the most frequently isolated uropathogen followed by *Candida* species (20.56%, 22/107), whereas, among gram-positive bacteria, *Enterococcus faecalis* (7.47%, 8/107) was the commonest, followed by *Staphylococcus aureus* (3.73%, 4/107) and *Staphylococcus saprophyticus* (2.8%, 3/107) (Figure 1).

Among *Candida* species, NAC species (59%, 13/22) outweigh the number of *Candida albicans* (41%, 9/22), with *Candida tropicalis* (22.72%, 5/22) being the commonest NAC. The distribution of *Candida* species and their antifungal susceptibility are tabulated in Table 1. All the *Candida* species were susceptible to voriconazole, whereas fluconazole resistance was observed in 31.81% (7/22) of *Candida* isolates, with a higher percentage in NAC species (22.72%, 9/22) than in *Candida albicans* (9.09%, 2/22). Out of seven fluconazole resistance isolates (by disk diffusion), the MIC of five isolates was ≥64 µg/ml (resistant), and that of two isolates was 16-32 µg/ml (susceptible dose-dependent).

**Table 1:** Distribution of Candida species isolated from pregnant women with significant bacteriuria and their antifungal susceptibility pattern *Excluding one intrinsic resistant *Candida krusei*

*Candida* species	Number (%)	Fluconazole		Voriconazole	
		Sensitive	Resistance	Sensitive	Resistance
Candida albicans	9 (40.9)	7 (31.81)	2 (9.09)	9	0
Non-albicans *Candida* species	13 (59.09)	7 (31.81)	5* (22.72)	13	0
Candida tropicalis	5 (22.72)	4	1	5	0
Candida parapsilosis	4 (18.18)	2	2	4	0
Candida glabrata	3 (13.63)	1	2	3	0
Candida krusei	1 (4.54)	0	1	1	0
Total	22	14 (63.63%)	7 (31.81%)	22 (100%)	0

The antimicrobial susceptibility pattern of all the bacterial uropathogens is depicted in Table 2, Table 3, and Table 4. All gram-negative bacilli were susceptible to carbapenems (imipenem and meropenem). Among *Enterobacterales*, 87.09% (54/62) of isolates and, among gram-positive cocci, 100% (12/12) of isolates were susceptible to nitrofurantoin. ESBL production was noted in 19.35% (12/62, eight *E. coli* and four *Klebsiella*) and AmpC production in 6.45% (4/62, all *E. coli*) of *Enterobacterales*. None of the gram-negative isolates were carbapenemase producers. All gram-positive cocci were resistant to penicillin. Methicillin resistance was observed in 50% (2/4) of *Staphylococcus* isolates and high-level aminoglycoside resistance (HLAR) in 75% (6/8) of *Enterococcus faecalis* isolates. However, all were susceptible to nitrofurantoin, vancomycin, and linezolid.

**Table 2:** Antimicrobial susceptibility pattern of Enterobacterales *Only for *E. coli* isolates NA: not applicable

Antimicrobial agent	*Escherichia coli* (n=46 (%))	*Klebsiella pneumoniae* (n=10 (%))	*Citrobacter koseri* (n=4 (%))	*Proteus mirabilis* (n=2 (%))
Ampicillin	20 (43.47)	0	0	0
Cefazolin	22 (47.82)	2 (20)	0	0
Gentamicin	38 (82.60)	6 (60)	2 (50)	2 (100)
Tobramycin	36 (78.26)	6 (60)	2 (50)	2 (100)
Amoxicillin/clavulanic acid	32 (69.56)	2 (20)	0	2 (100)
Piperacillin/tazobactam	42 (91.30)	10 (100)	4 (100)	2 (100)
Cefuroxime	30 (65.21)	4 (40)	2 (50)	1 (50)
Cefoxitin	36 (78.26)	8 (80)	4 (100)	2 (100)
Cefotaxime	34 (73.91)	6 (60)	4 (100)	2 (100)
Cefepime	36 (78.26)	6 (60)	4 (100)	2 (100)
Imipenem	46 (100)	10 (100)	4 (100)	2 (100)
Meropenem	46 (100)	10 (100)	4 (100)	2 (100)
Amikacin	40 (86.95)	8 (80)	4 (100)	2 (100)
Ciprofloxacin	40 (69.56)	5 (60)	2 (50)	1 (50)
Levofloxacin	34 (73.91)	5 (60)	4 (100)	1 (50)
Ceftazidime	34 (73.91)	6 (60)	4 (100)	2 (100)
Nitrofurantoin	42 (91.30)	8 (80)	4 (100)	0
Cotrimoxazole	36 (78.26)	6 (60)	4 (100)	0
Fosfomycin*	46 (100)	NA	NA	NA

**Table 3:** Antimicrobial susceptibility pattern of nonfermenter isolates

Antimicrobial agent	*Pseudomonas aeruginosa* (n=4 (%))	*Acinetobacter baumannii* (n=4 (%))
Ampicillin/sulbactam	-	4 (100)
Piperacillin/tazobactam	4 (100)	0
Ceftazidime	2 (50)	0
Cefepime	4 (100)	2 (50)
Cefotaxime	-	0
Aztreonam	4 (100)	-
Imipenem	4 (100)	4 (100)
Meropenem	4 (100)	4 (100)
Gentamicin	2 (50)	0
Tobramycin	2 (50)	0
Amikacin	4 (100)	2 (50)
Ciprofloxacin	2 (50)	0
Levofloxacin	2 (50)	0
Minocycline	-	4 (100)
Tetracycline	-	4 (100)
Cotrimoxazole	-	4 (100)

**Table 4:** Antimicrobial susceptibility pattern of gram-positive cocci *Surrogate marker for MRSA ^†^MIC by E strip was in the susceptible range MRSA: methicillin-resistant *Staphylococcus aureus; *MIC: minimum inhibitory concentration

Antimicrobial agent	*Enterococcus faecalis* (n=8 (%))	*Staphylococcus aureus* (n=4 (%))
Penicillin	2 (25)	0
Ampicillin	4 (50)	-
Cefoxitin* (oxacillin)	-	2 (50)
Gentamicin	-	3 (75)
Gentamicin high level	6 (75)	-
Streptomycin high level	6 (75)	-
Ciprofloxacin	4 (50)	1 (25)
Levofloxacin	4 (50)	1 (25)
Vancomycin	8 (100)	4 (100)^†^
Linezolid	8 (100)	4 (100)
Tetracycline	8 (100)	4 (100)
Nitrofurantoin	8 (100)	8 (100)
Cotrimoxazole	4 (50)	2 (50)
Fosfomycin	8 (100)	-

## Discussion

UTI in pregnancy needs special attention as it can progress to complications if not treated on time. Further emergence of drug resistance adds to the problem. Urine culture and antimicrobial susceptibility testing are necessary in pregnancy for early diagnosis and prompt treatment.

In the present research, the prevalence of UTI in pregnancy was 26.75% (107/400), which is comparable with the study conducted by Sabharwal (24%) [17]. However, Rizvi et al. [9] reported a higher (51.2%) prevalence, and David et al. [18] reported a lower (9.49%) prevalence. Moreover, we observed a higher prevalence of ASB (58.87%) than symptomatic UTI (41.12%). A similar finding of relatively higher ASB was also noted by Kerure et al. [19] (65%>35%), Rizvi et al. [9] (74.8%>25.2%), and Sabharwal [17] (75%>25%).

*Escherichia coli* was the commonest uropathogen isolated (42.99%). Most other studies also reported *E. coli* as the commonest uropathogen [4,6,9,17-19]. In contrast, Patnaik et al. [20] found *Klebsiella* species to be the commonest isolate. All the gram-negative bacilli were susceptible to carbapenems, which is similar to the study conducted by Rizvi et al. [9], Sabharwal [17], and Patnaik et al. [20]. However, among *Enterobacterales*, 19.35% of the isolates were ESBL producers and 6.45% were AmpC producers. Rizvi et al. [9] reported that 47% of *E. coli* and 36.9% of *Klebsiella pneumoniae* isolates were ESBL producers and 10% of *Enterobacterales* were found to be AmpC producers. Sabharwal [17] reported that 45% of *E. coli* and 40% of *Klebsiella* species were ESBL producers. David et al. [18] reported 30% of *E. coli* as ESBL producers.

Among gram-positive cocci, the commonest isolate was *Enterococcus faecalis* (7%), comparable with the study by David et al. [18] (13%). In contrast, the commonest gram-positive cocci reported were *Staphylococcus aureus* (9%) by Kerure et al. [19] and coagulase-negative staphylococci (6.4%) by Rizvi et al. [9]. In the present study, 50% of staphylococcal isolates were methicillin-resistant, and 75% of enterococcal isolates were HLAR. However, all were susceptible to nitrofurantoin, vancomycin, and linezolid. Similarly, Rizvi et al. [9] reported 34% methicillin-resistant *Staphylococcus aureus* (MRSA) and 30% HLAR enterococci. Sabharwal [17] observed more than one-third of staphylococcal isolates as methicillin-resistant.

Candiduria was observed in 20.56% of cases with a relatively higher percentage of NAC species. Similar findings were reported by other authors [7,21,22]. Overall, fluconazole resistance was 31.81%; however, the percentage was higher in NAC species (22.72%) than in *Candida albicans* (9.09%). Similarly, an increasing trend was observed by other researchers too [7,21,22].

Limitations

Production of beta-lactamases (ESBL and AmpC) was determined by phenotypic methods, and antifungal susceptibility was performed by disk diffusion and E-test only due to lack of funding.

## Conclusions

UTIs in pregnancy are caused by a spectrum of both gram-negative bacilli and gram-positive cocci. There is an increased prevalence of ASB in pregnancy, which necessitates the screening of pregnant females for UTI using culture and antimicrobial susceptibility testing. There is the emergence of drug resistance in uropathogens, with an increasing prevalence of ESBL, AmpC, and MRSA among community-acquired UTIs also. This limits the choice of antimicrobials for treatment in pregnancy.

Moreover, there is a rise of both *Candida albicans* and NAC species as uropathogens, with the emergence of fluconazole resistance. Urine culture and antifungal susceptibility testing are of utmost importance for identifying the *Candida* species and its susceptibility pattern. Hence, irrespective of urinary symptoms, urine culture with antimicrobial susceptibility testing during pregnancy should be routinely done for early diagnosis and prompt treatment.
